# Latent classes and related predictors of demand for home-and community-based integrated care for older Chinese adults

**DOI:** 10.3389/fpubh.2023.1109981

**Published:** 2023-06-23

**Authors:** Zhenyu Wang, Zhihan Liu

**Affiliations:** ^1^School of Government, Sun Yat-sen University, Guangzhou, Guangdong, China; ^2^School of Public Administration, Central South University, Changsha, Hunan, China

**Keywords:** integrated care, home-based care, community-based care, older people, latent profile analysis

## Abstract

**Background:**

Home-and community-based integrated care has been developing rapidly in China in recent years. However, empirical research on the demand from older people is insufficient. Most studies have failed to identify or differentiate the heterogeneity of older people, resulting in poor understanding of their needs and fragmentation of services. This study seeks to identify latent classes of demand for home-and community-based integrated care among older Chinese adults and the predictors that differentiate these demands.

**Methods:**

From January to March 2021, a questionnaire was administered to older people (aged ≥60 years) in community-based service centers for older people in six districts of Changsha City, Hunan Province. Participants were selected through purposive and incidental sampling. Latent profile analysis was used to categorize older people’s demand for home-and community-based integrated care. By extending Andersen’s behavioral model of health service use, and running multinomial logistic regression analyses, we explored which factors influenced the latent classes of demand.

**Results:**

A total of 382 older people were included in the analyses: 64.4% were women and 33.5% were aged 80–89. The demand from older people for home-and community-based integrated care was classified into four latent classes: high health and social interaction demand (30% – 115/382); high comprehensive demand (23% – 88/382); high care service demand (26% – 100/382), and high social participation and low care demand (21% – 79/382). Taking this last class as the reference group, the other three latent classes differed significantly in the factors of predisposition, enabling, need, and perception of aging.

**Conclusion:**

The demand from older people for home-and community-based integrated care is multifaceted and heterogeneous. Services for older people should be designed with different sub-models of integrated care.

## 1. Introduction

China has an aging society. The World Health Organization (WHO) estimates that, by 2050, 35.1% of China’s population will be aged 60 and over (1). By 2030, the proportion of empty nesters (those with no children to care for), living alone or only with their spouse, is expected to increase to 90% (2). The trend of socializing family care responsibilities implies that care for older individuals is increasingly being provided by formal carers outside the immediate family. However, the current social pension and public service systems in China are not yet sound. Aging tends to bring a decline in physical functions and an increase in chronic diseases, which may make older people increasingly dependent on medical resources, in turn increasing the burden on their caregivers (3).

It is imperative, therefore, that the limited nursing and medical resources are prioritized to meet the demand from older people for quality care and services. “Aging in place” is seen as an advantage that allows older people to be cared for in a familiar place, and is generally welcomed by older people (4). However, with many family members now relocating for work, parents who stay in their own home may be left unattended. In 2013, as a response to this issue, the Chinese government published “Several Opinions on Accelerating the Development of the Older People Service Industry” (5). This set out a clear vision, and a 2030 target, for a home-and community-based care system that is functional, moderate in scale, and covers both urban and rural areas.

Home-and community-based care should be the future direction for meeting the needs of older people in China. At the same time, however, with current economic and social changes, family support is weakening and the demand for community services for older people is increasing. This creates opportunities for innovative models of older people care (6). Integrated care has emerged as an effective way to meet the complex needs of older people with chronic conditions (7), replacing high-cost hospital or residential care with more cost-effective, more sustainable and higher quality home and community-based care (3).

In China, *Yiyang Jiehe* (医养结合) has become synonymous with, and the main manifestation of integrated care for older people (8). *Yiyang Jiehe* combines medical and nursing care resources in a way that complement each other. Doctors, nurses, caregivers, social workers, community staff and other relevant professions provide services that include medical care, basic living care, rehabilitation and health care, cultural and recreational activities and spiritual comfort for older people in different scenarios, such as home, community, nursing home and hospital, and through different modes or forms, to maintain the physical and mental health of older people and improve their independence and social participation. After nearly a decade of development, four primary modes of *Yiyang Jiehe* have been created in China: hospital care in nursing homes, nursing care in hospital, contract cooperation between hospitals and nursing homes and community-based adult services (CBAS) integrated with medical care (9). This fourth mode uses an integrated care approach to improve the quality of home and community care services for older people living permanently in the community or at home, and this is the focus of this study.

Academic consensus is still lacking, however, in the definition and detail of service content (10). The various translations of *Yiyang Jiehe* by Chinese academics include “medical–nursing combination/combined” (11, 12), “medical and elderly care combination” (13), “integrating pension service with medical service” (14), “integrated medical and nursing care” (15), and “combination of medical care and pension” (16). To facilitate understanding by Chinese and foreign readers, this paper translates *Yiyang Jiehe* as “home-and community-based integrated care.” This refers to a government-led service model based in the home and community that aims to integrate older people’s care and medical services, and develop various channels of older people services, so that their later years can be spent in a familiar environment (17).

China is relatively late in pursuing this type of integration, and existing studies have focused primarily on promotion of the integrated care model, clarification of concepts, and construction of institutional frameworks (11, 18). Meanwhile, some empirical studies in China have failed to identify or differentiate the heterogeneity of older people’s needs (11, 17), There have also been a study that distinguished latent categories of older people’s care needs but did not focus on the topic of home-and community-based integrated care (19). Older people living in the community vary significantly in the type, content, quantity and nature of the services they require. The fragmentation of care services is not well addressed when the needs of older people are assessed in a homogenous manner or not truly understood, leading to the wasteful and inappropriate deployment of home-and community-based social care models (20).

**Figure 1:**
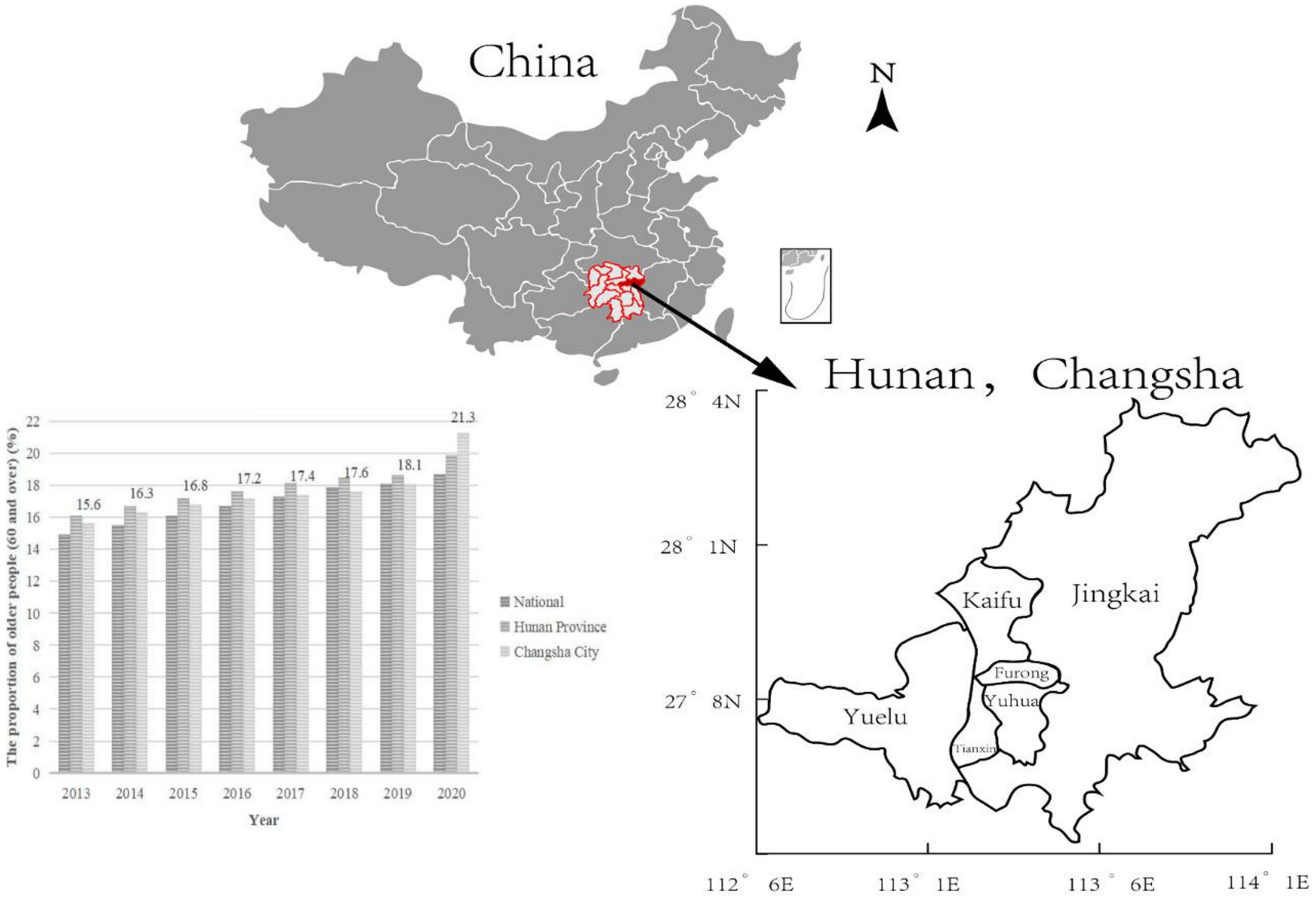
Population aging trends from 2013 to 2020, and the geographical location of Changsha and its six districts in Hunan.

In 2013, China made its first explicit proposal to “actively promote the *Yiyang Jiehe*” model and services (5). In south-central China, Hunan Province is one of the provinces most affected by aging. According to the 7th census of China, 19.88% of the population of Hunan is aged 65 or over, which exceeds the 14% threshold to be classified as a “deeply aging society” in the United Nations definition. Changsha City, the provincial capital, has the largest permanent population in Hunan. As early as 2016, Changsha’s population aged 60 years and above reached 17.2%, ranking second among central provincial capital cities (21). From 2013 to 2020, Changsha’s aging population (60 and above) jumped from 15.60 to 21.30%. In comparison, the national figure for 2020 was 18.7% and for Hunan was 19.88% [see Figure 1, data from the China Statistical Yearbook (22) and Hunan Statistical Yearbook (23)]. This severe level of aging in the population will undoubtedly put enormous pressure on social and home care services in Changsha, resulting in huge demand for the development of integrated care services.

**Figure 2:**
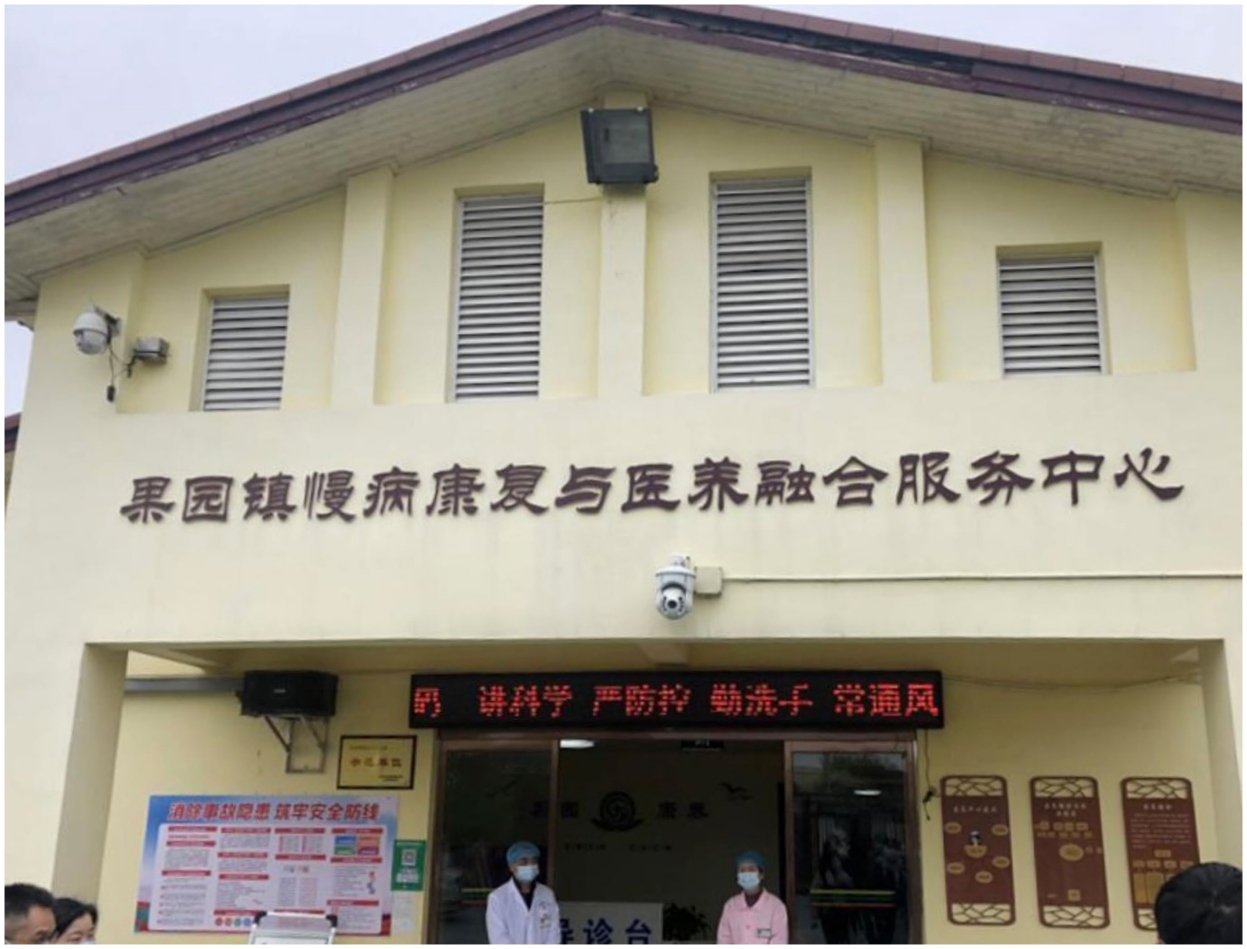
The “yiyangjiehe” Centre in Changsha County, Guoyuan Township.

As a national pilot city for *Yiyang Jiehe*, Changsha has made some progress. By 2019, 272 urban home-and community-based care service centers had been established in the city, evenly distributed across the six districts (see Figure 1). These centers have entered contracts with integrated tertiary hospitals and formed partnerships with community hospitals. They are also equipped with community family doctors. They provide people aged 60 and over with free regular physical examinations, family doctor services, referrals, home nursing care, and other services. Some districts have innovated in their implementation of *Yiyang Jiehe.* For example, Orchard Town, Jingkai District (Changsha County) has established a *Yiyang Jiehe* center (see Figure 2) and adopted the “1 + X” family doctor sign pattern: “1” denotes a general practitioner, while “X” includes a public health doctor, nurse, manager and higher-level hospital specialists. The aim is manage “the last mile” of medical and health services for older people, with facilities located within one mile of the community (24). However, Changsha City faces multiple challenges to successfully deliver integrated care services and meet the complex needs of older people. These include an inadequate assessment system for older people services, lack of professional caregivers, and insufficient information-sharing (25).

As a person-centered research method, latent class analysis (LCA) or latent profile analysis (LPA) enables the individual needs of older people to be differentiated and their heterogeneity to be understood (26). For example, Fu and Chui used LCA to identify heterogeneity in the needs of older people for home-and community-based care services (27). They identified three groups—high, moderate, and low needs—and explored how living arrangements and filial piety influenced each of these groups. Filial piety is a concept that originates from Confucianism and refers to the virtue of respect, care, and obedience that one has toward parents and older generations. In an LCA of older people in Urumqi, Xinjiang province, the needs of older people were divided into three subgroups: medical care demand, comprehensive demand and no demand (28). More recently, Wang conducted an LCA of 6,364 older Chinese people based on four demand dimensions: economic security, living care, health care and spiritual comfort, and the study found that, in terms of needs, older people could be classified into four groups: high health care demand, comprehensive high demand, healthy multi-demand, and low demand (19).

Meanwhile, a scoping review has shown that demographic factors (age, gender, education and occupation), individual factors (self-rated health status, economic status, number of chronic diseases and knowledge of integrated care), family factors (number of children and family relationships), and community factors (service quality, infrastructure facilities and neighborhood relations) are the main determinants of older people’s demand for integrated care in community settings (29). Overall, research suggests that the factors influencing older people’s demand for integrated care services are wide-ranging and fragmented, with complex interrelationships. The promotion and improvement of care models is an interactive process, making it especially important and relevant to examine the influences of demographic, individual, family and community factors through an LCA approach and a classification of older people’s needs.

To date, however, empirical research on the integrated care model in China has been lacking, paying little attention to the willingness of older people to engage with integrated care, and to whether the services currently provided effectively meet their requirements (11, 18). As older people are heterogeneous in terms of health status and psychological needs, what they need from home-and community-based integrated care is influenced by various factors around their daily life, medical care, emotional wellbeing, social participation, welfare and so on.

Most research in China has been limited to dichotomous variables. Further limitations include vagueness around the sources of and methods for selecting variables, and insufficient investigation into the factors influencing latent classes of older people’s needs. In addition, research in China has lacked a theoretical analysis framework for the choice of or demand for integrated care services (30). This paper, therefore, seeks to address these issues, by adopting Andersen’s behavioral model of health service utilization (ABMHSU) (31) as the framework for theoretical analysis, and investigating the key factors that influence the demand for home-and community-based integrated care.

The needs and wishes of older people, as the main target group of integrated care services, play a decisive role in the development of *Yiyang Jiehe* (13). Therefore, after constructing a reasonable assessment tool and collecting data, this study explores the heterogeneity of demand for home-and community-based integrated care for older people and related predictors. This study aims to provide insights into the current needs of older people in the community and make recommendations for the development of home-and community-based integrated care for older people in China.

## 2. Methods

This study was approved by the Clinical Medical Ethics Committee of Xiangya Hospital, Central South University (No. 202011184), and all study participants gave informed consent and participated voluntarily.

### 2.1. Data and sample

For this cross-sectional study, a questionnaire survey was conducted from January to March 2021 through a combination of purposive and incidental sampling techniques. The target population was older adults in home-and community-based senior care centers in six focal districts of Changsha City: Kaifu, Furong, Yuhua, Yuelu, Tianxin, and Jingkai. The questionnaire was administered through face-to-face interviews conducted by the researcher in our team. The researcher completed and checked the questionnaire to avoid difficulties or errors in completion by participants. To be eligible to participate, an individual had to meet all the following criteria: (1) aged 60 and above; (2) resident in any of the six focal districts for at least 1 year; (3) living in the community and at home, rather than in a nursing home; (4) able to give informed consent and participate voluntarily; and (5) able to communicate normally, understand the questions asked, and accurately express their true thoughts. Individuals with hearing or cognitive impairment or serious mental illness were not eligible to participate. We also excluded any participants who were not cooperative during the interview or withdrew from the survey. In total, 382 valid questionnaires were distributed and collected, with a 100% return rate.

### 2.2. Measures

Based on mixed research methods and the principle of triangulating evidence, the structured questionnaire was designed based on semi-structured interviews with older people (9), a scoping review (29), advice from experts, and how older people’s care was provided in Changsha. The questionnaire comprised two main parts: the first covered older people’s demand for home-and community-based integrated care (Part 1, 30-items), while the second covered the influencing factors, based on ABMHSU (Part 2, 30-items). The questionnaire is set out in Supplementary Appendix 1.

To ensure the questionnaire had good test reliability and provided a valid survey, several prerequisites had to be satisfied, including exploratory factor analysis (EFA) (Kaiser-Meyer-Olkin (KMO) and Bartlett’s tests) and confirmatory factor analysis (CFA) (32). For EFA, the KMO test estimates sampling adequacy that expresses the proportion of variance among the investigated variables that could be a common variance. The recommended KMO value is higher than 0.5 (33). Bartlett’s test was performed to test the correlation between variables in a correlation matrix, i.e., to test whether the variables were independent of each other (34). Secondly, CFA is commonly used to test the applicability of the structural validity of measurement instruments and the model fit through indicators such as relative chi-square (CMIN/DF, where a smaller value is better), root-mean-square error of approximation (RMSEA: <0.08 acceptable, <0.05 excellent), goodness-of-fit index (GFI), adjusted goodness-of-fit index (AGFI), Tucker-Lewis index (TLI), incremental fit index (IFI) and comparative fit index (CFI) (35), and the evaluation of GFI, AGFI, TLI, IFI and CFI values all follow the same criteria: >0.85 acceptable, >0.95 excellent (33, 36). In addition, construct validity was assessed by average variance extracted (AVE: >0.5 acceptable) and composite reliability (C.R.: >0.7 excellent) (37). It is important to note that Part 1 of the questionnaire was completely self-administered, whereas Part 2 was mainly based on the well-established ABMHSU and included many demographic variables and non-Likert five-point questions. Therefore, only the items of Part 1 were tested for EFA and CFA.

Generally, a Cronbach’s alpha coefficient of 0.7 or above makes a questionnaire more reliable (33). The whole questionnaire had an overall Cronbach’s alpha coefficient of 0.871 (Part 1 Cronbach’s α = 0.901; Part 2 Cronbach’s α = 0.722). EFA showed that the KMO = 0.74, Sig < 0.001. CFA of model fit was assessed using the following fit indices: CMIN/DF = 1.503, RMSEA = 0.036, GFI = 0.903, AGFI = 0.878, CFI = 0.857, TLI = 0.859, and IFI = 0.855; (C.R.) = 0.976 and AVE = 0.581. Therefore, the questionnaire and its model constructed for this study had a good degree of fit, reliability and validity.

#### 2.2.1. Demand for home-and community-based integrated care

Part 1 of the questionnaire contained 30 questions across seven dimensions: basic life services, medical–nursing services, rehabilitation services, psycho-spiritual support services, health education services, social participation and welfare services, and social aid services. Participants responded to each item on a Likert five-point scale (1 = very unneeded, 5 = very needed). Basic life services were examined through eight items: preparing meals, housekeeping, on-site service (home visits), shopping, transport, hotline service, day care and safety. The six items of medical–nursing services covered disease treatment, home nursing care, medical transport, preventive care, Chinese medicine service and injections. Rehabilitation services were examined through four items: massage, acupuncture, physical therapy and rehabilitation guidance. The three items of psycho-spiritual support services covered spiritual solace, psychological counseling, and company. Health education services were examined through four items: health counseling, health guidance, first aid training, and life skills training. The three items of social participation services covered provision of exercise venues and opportunities, voluntary activities, and cultural and entertainment services. Finally, social aid services were examined through two items: legal aid and dispute mediation. See Table 1 for a description of home-and community-based integrated care.

**Table 1:** An overview of home-and community-based integrated care for older adults.

Demand dimensions	Description	Mode of funding	Service site	Types of providers
Basic life services	Including basic daily services such as accommodation, providing home-delivered and communal meals, providing adult day care, helping clean homes and shopping and ensuring that they can move safely, etc.	National special subsidy, out-of-pocket expenses for older people and their families, insurance (medical insurance, long-term care insurance, commercial insurance), social capital or PPP (public-private partnership) mode	Home (on-site service) or community older people’s care centers	Governments, older people’s care companies, healthcare institutions (community health centers, community older people’s care facilities), community health workers (CHWs), NGOs (non-government organizations) or NPOs (non-profit organizations)
Medical–nursing services	Mainly community-based primary health care services covering disease diagnosis and treatment, regular physical check-ups, Chinese medicine services, chronic disease management, bidirectional referral and home visits by family doctors, etc.			
Rehabilitation services	Using traditional Chinese medicine therapies such as massage, acupuncture, physical therapy, and rehabilitation guidance to help older people restore their physical functions			
Psycho-spiritual support services	Helping older people to cope with their problems and avoid depression and loneliness through psychological counseling, companionship, chatting, etc.			
Health education services	Promoting health literacy among the older adult through health education, life skills training or health lectures			
Social participation services	Providing recreational and interactive services to help older people return to society and form their own social networks			
Social aid services	Mainly legal aid and dispute mediation			

#### 2.2.2. Influencing factors based on an extended Andersen’s behavioral model of health service use

**Figure 3:**
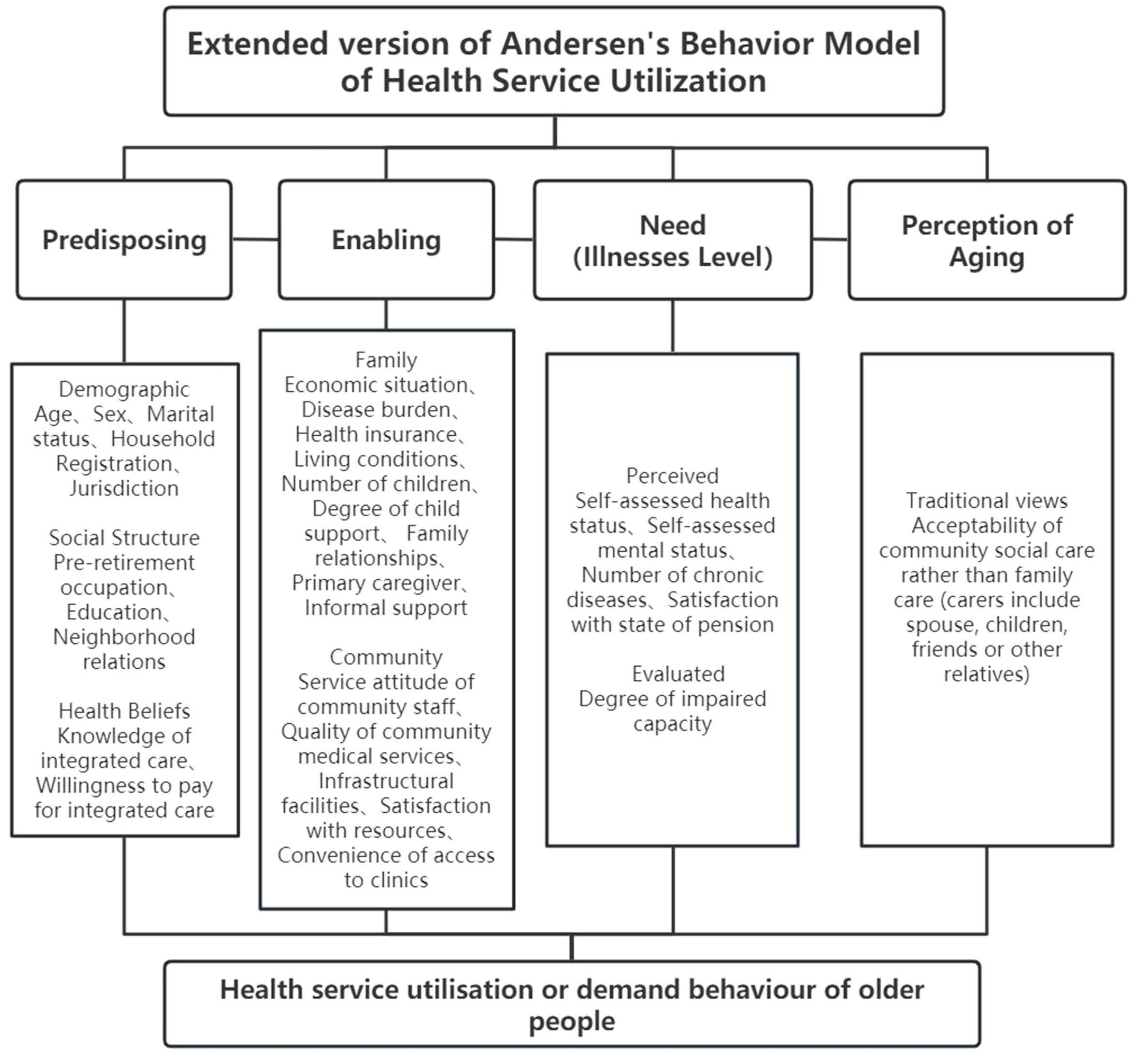
Theoretical framework of factors potentially influencing older people’s demand for home-and community-based integrated care in older people, based on the extended ABMHSU.

Andersen’s behavioral model of health service use (ABMHSU) is an internationally authoritative model for health service research. It suggests that, in deciding whether to use health services, individuals are influenced by three dimensions: predisposition, enabling and need factors (31). Policymakers and health service researchers can use ABMHSU to optimize health service use and reform health service systems (38). This model has been used in China and elsewhere to study various aspects of older people’s care and health service use, including willingness to age at home and screening for chronic diseases (39–41). As ABMHSU was developed in the West, this model needs to be revised to fit the Chinese context. Studies have shown that a revision of the Andersen model to construct a system of indicators is suitable as a theoretical framework for analyzing the factors influencing preferences for older people’s perception of aging and aged care mode choice (42). An important innovation in our study is the addition of factor of perception of aging, which improves the indicator system and theoretical analysis framework (including 30 questions, see Figure 3). For the need factors, older people’s degree of impairment or capacity was measured using the Standardized Scale for the Assessment of the Abilities of Older People (Trial) on a Likert four-point scale (1 = heavily impaired, 2 = moderately impaired, 3 = slightly impaired, 4 = unimpaired) (43). This scale was developed in line with the WHO International Classification of Functioning, Disability and Health (ICF), the Activities of Daily Living Scale (ADLs) and the Instrumental Activities of Daily Living Scale (IADLs). This instrument was tailored according to the characteristics of older people’s care in China. It contains 27 items across three dimensions: the ability to perform the activities of daily living; mental state and social participation, and perceptual and communication abilities.

### 2.3. Statistical analysis

The data was entered via EpiData 3.1 and subsequently imported into SPSS 21.0 for collation. The processed data was analyzed in Mplus 8.0 for LPA.

#### 2.3.1. Latent profile analysis

LPA is an extension of LCA, which is centered on the individual. It ensures that the between-group variance of the classification results is maximized and the within-group variance minimized. It is a appropriate method for studying heterogeneity in older populations (44). The main reference indicators for model-fit include log likelihood (LogLik), Akaike’s information criterion (AIC), Bayesian information criterion (BIC), adjusted BIC (aBIC), Lo–Mendell–Rubin (LMR) and the bootstrap likelihood ratio test (BLRT). In general, smaller values of AIC, BIC and aBIC mean a better fit. If the *p*-values of the LMR and the BLRT are significant (*p* < 0.05), this indicates the target profile solution (n) fits better than a solution with one fewer profile (*n*−1) (45).

#### 2.3.2. Descriptive statistics, one-way and multinomial logistic regression analyses

SPSS 21.0 was used to conduct one-way and multinomial logistic regression analyses of the distribution of each latent class across the independent variables (a total of 30 items on the influencing factors of predisposition, enabling, need, and perception of aging based on the extended ABMHSU). A chi-square ( $x^{2}$ ) test was conducted for the categorical variables and a one-way analysis of variance was conducted for the continuous variables. The measurement data were expressed as mean ± standard deviation (*M* ± SD); the count data were described using frequency (n) and rate (%). The threshold for statistical significance was set at *p* < 0.05.

## 3. Results

The statistics for both the total score and the dimensions of older people’s demand were normally distributed. Therefore, *M* ± SD can be used for statistical description. For the full sample of 382 older people, total demand was 85.39 (±18.745), which is higher than the theoretical total score of 75 (=2.5 × 30), indicating high overall demand. Looking at the mean scores of the seven dimensions, the demand from older people for social participation services (3.38 ± 1.087) and medical–nursing services (3.13 ± 0.993) were high; demand for rehabilitation services (2.99 ± 1.267) and basic life services (2.83 ± 0.995) were relatively high; demand for health education services (2.71 ± 1.141) was moderate, and demand for psycho-spiritual support services (2.45 ± 1.279) and social aid services (1.82 ± 1.155) were low.

### 3.1. Latent profile analysis and model fit

As reported in Table 2, the LogLik AIC, BIC, and aBIC values decreased as the number of latent classes increased. Although the AIC, BIC, and aBIC values were optimal for six classes, the LMR and BLRT values supported retaining four classes (*p* < 0.001) rather than six (*p >* 0.05), and the differences between the respective AIC, BIC and aBIC values for four and six classes were small. Based on the combined results for these indicators, the four-class model appeared optimal.

**Table 2:** Fit indicators for different latent profile models.

Model	LogLik	Npar	AIC	BIC	aBIC	LMR	BLRT	Class probability
1	−4108.30	14	8244.61	8299.84	8255.42	–	–	1
2	−3952.99	22	7949.98	8036.78	7966.98	<0.001	<0.001	0.77/0.23
3	−3895.19	30	7850.37	7968.74	7873.55	0.123	<0.001	0.37/0.22/0.41
4	−3850.19	38	7776.39	7926.31	7805.74	<0.001	<0.001	0.21/0.30/0.26/0.23
5	−3819.86	46	7731.72	7913.21	7767.26	0.331	<0.001	0.21/0.29/0.05/0.26/0.19
6	−3785.06	54	7678.13	7891.18	7719.85	0.999	1.000	0.24/0.19/0.11/0.26/0.06/0.14

LogLik, Log likelihood test; Npar, Number of free parameters; AIC, Akaike information criterion; BIC, Bayesian information criterion; aBIC, adjusted Bayesian information criterion; LMR, Lo-Mendell-Rubin likelihood ratio test; BLRT, bootstrap likelihood ratio test.

### 3.2. Naming of latent classes

**Figure 4:**
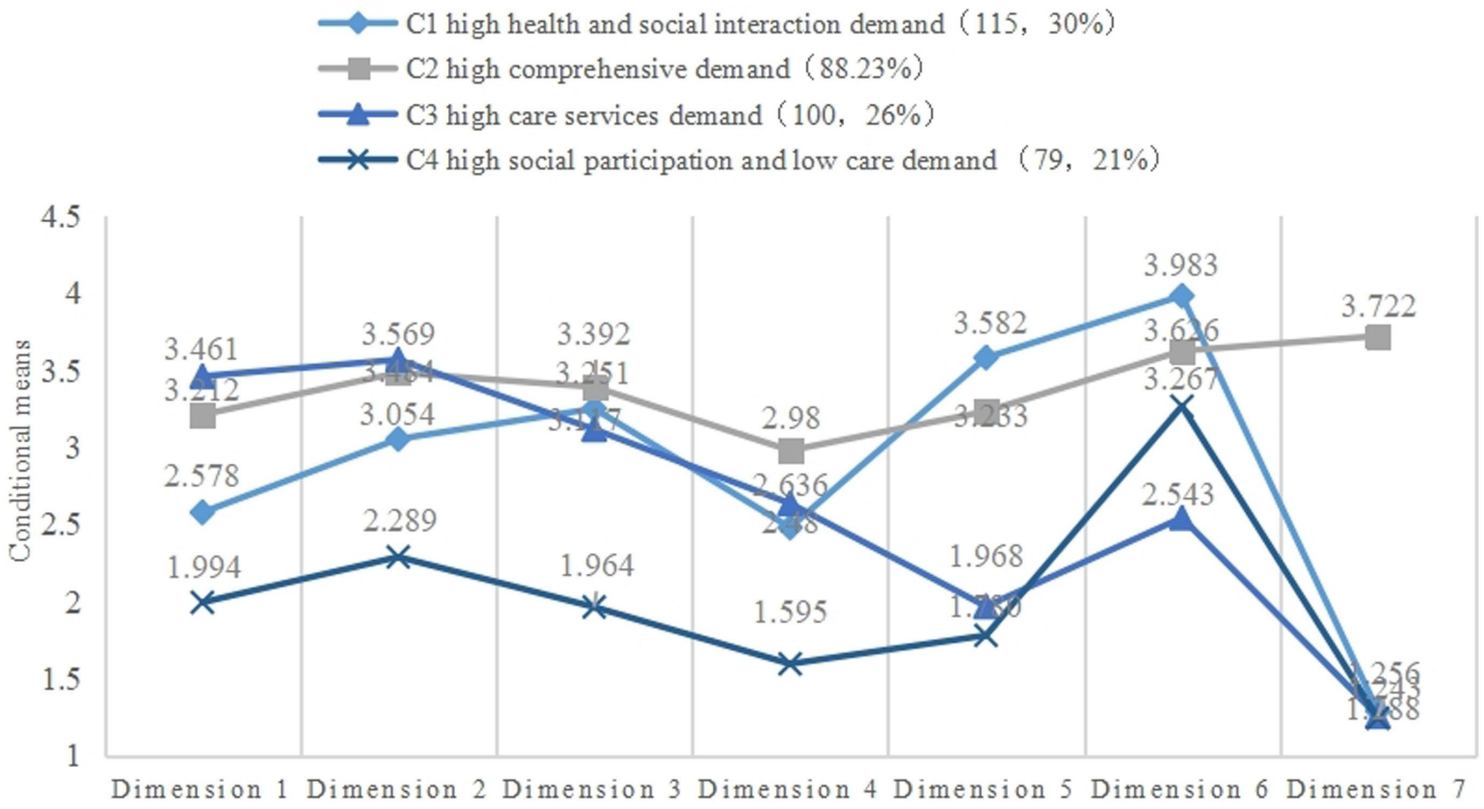
The extent of demand across seven dimensions in the four latent classes. Dimension 1, basic life services; Dimension 2, medical–nursing services; Dimension 3, rehabilitation services; Dimension 4, psycho-spiritual support services; Dimension 5 health education services; Dimension 6, social participation and welfare services; Dimension 7, social aid services.

Using the selected model, conditional means were obtained for the four latent classes on seven dimensions (see Figure 4). Class 1 (C1) had high health and social interaction demand (115, 30%), denoted by the highest conditional means for health education and social participation, as well as relatively high demand for rehabilitation services and medical–nursing services. Class 2 (C2) had high comprehensive demand (88, 23%), exhibiting relatively high conditional means on all seven dimensions, albeit with a slightly lower conditional mean on basic life services relative to C1. Class 3 (C3) had high care demand (100, 26%), denoted by the highest scores for basic life services and medical care services, as well as high scores for rehabilitation services. Finally, Class 4 (C4) had high social participation and low care demand (79, 21%), scoring highly for social participation services only, and with mean scores below 2.5 for the other six dimensions. Except for C2, all latent classes scored extremely low for social aid services, which may be due to the low income of older people in C2.

### 3.3. Descriptive statistics and one-way ANOVA

#### 3.3.1. Predisposing factors

The sample contained more women than men (64.4% vs. 35.6%), and the majority (209, 54.7%) had a spouse. The largest age group was 80–89 years (128, 33.5%), followed by 60–69 (115, 30.1%) and 70–79 (107, 28.0%). Regarding education level, the smallest group was no or little literacy (21, 5.5%). 303 (79.3%) were registered as urban and 79 (20.7%) rural. The differences of age, gender, marital status, jurisdiction, neighborhood relations, knowledge of integrated care and willingness to pay for integrated care between the four latent classes were statistically significant (*p* < 0.05), while household registration (urban or rural), pre-retirement occupation, and education level were not statistically significant (*p* ≥ 0.05). A greater proportion of older people in C1 and C2 were aged 60–69, and more older people in C3 were aged 80–89; women were more likely to be in C1 than men, and a greater proportion of older people with spouses were in C1, C2, and C4 than those without spouses. Table 3 presents the full results.

**Table 3:** Predisposing factors of latent classes (*N* = 382).

Variables	C1^a^ (*n* = 115, 30%)	C2^b^ (*n* = 88, 23%)	C3^c^ (*n* = 100, 26%)	C4^d^ (*n* = 79, 21%)	*x*^2^/F	Scheffe *post-hoc*
Age					37.089^***^	–
60–69	37 (32.2%)	37 (42.0%)	14 (14.0%)	27 (34.2%)		
70–79	39 (33.9%)	26 (29.5%)	20 (20.0%)	22 (27.8%)		
80–89	32 (27.8%)	20 (22.7%)	52 (52.0%)	24 (30.4%)		
90–99	7 (6.1%)	5 (5.7%)	14 (14.0%)	6 (7.6%)		
Sex					11.661^**^	–
Male	27 (23.5%)	37 (42.0%)	37 (37.0%)	35 (44.3%)		
Female	88 (76.5%)	51 (58.0%)	63 (63.0%)	44 (55.7%)		
Marital status					18.380^***^	–
With spouse	63 (54.8%)	59 (67.0%)	38 (38.0%)	49 (62.0%)		
Without spouse (including unmarried, separated or widowed)	52 (45.2%)	29 (33.0%)	62 (62.0%)	30 (38.0%)		
Household registration					6.550	–
Urban	86 (74.8%)	73 (83.0%)	86 (86.0%)	58 (73.4%)		
Rural	29 (25.2%)	15 (17.0%)	14 (14.0%)	21 (26.6%)		
Jurisdiction					38.729^**^	–
Kaifu	10 (8.7%)	13 (14.8%)	23 (23.0%)	11 (13.9%)		
Furong	9 (7.8%)	9 (10.2%)	11 (11.0%)	5 (6.3%)		
Yuhua	32 (27.8%)	18 (20.5%)	35 (35.0%)	14 (17.7%)		
Yuelu	37 (32.2%)	18 (20.5%)	9 (9.0%)	18 (22.8%)		
Tianxin	13 (11.3%)	11 (12.5%)	13 (13.0%)	17 (21.5%)		
Jingkai	14 (12.2%)	19 (21.6%)	9 (9.0%)	14 (17.7%)		
Pre-retirement occupation					24.137	–
Public institution staff	39 (33.9%)	29 (33.0%)	50 (50.0%)	27 (34.2%)		
Corporate staff	30 (26.1%)	26 (29.5%)	25 (25.0%)	22 (27.8%)		
Technical staff	4 (3.5%)	9 (10.2%)	11 (11.0%)	7 (8.9%)		
Self-employed	8 (7.0%)	3 (3.4%)	5 (5.0%)	5 (6.3%)		
Farmer	23 (20.0%)	17 (19.3%)	6 (6.0%)	12 (15.2%)		
Freelancer	11 (9.6%)	4 (4.5%)	3 (3.0%)	6 (7.6%)		
Education					15.486	–
No or little literacy	6 (5.2%)	3 (3.4%)	9 (9.0%)	3 (3.8%)		
Primary school	30 (26.1%)	14 (15.9%)	29 (29.0%)	24 (30.4%)		
Junior high school	28 (24.3%)	24 (27.3%)	15 (15.0%)	21 (26.6%)		
High school or technical secondary school	27 (23.5%)	23 (26.1%)	24 (24.0%)	15 (19.0%)		
Junior college	7 (6.1%)	9 (10.2%)	5 (5.0%)	5 (6.3%)		
Bachelor and above	17 (14.8%)	15 (3.9%)	18 (18.0%)	11 (13.9%)		
Neighborhood relations	4.09 ± 0.812	3.81 ± 0.957	3.84 ± 0.748	4.01 ± 0.809	2.633^*^	a > d > c, a > d > b
Knowledge of integrated care	2.72 ± 1.460	2.73 ± 1.293	3.22 ± 1.315	2.43 ± 1.375	5.272^**^	c > b > a, c > a > d
Willingness to pay for integrated care	3.66 ± 0.936	3.78 ± 1.077	3.92 ± 0.907	3.48 ± 1.011	3.231^**^	c > b > a, c > a > d

*, **, and *** denote significance levels of 5, 1, and 0.1%, respectively. C1, high health and social interaction demand; C2, high comprehensive demand; C3, high care service demand; C4, high social participation and low care demand. ^a–d^denote C1, C2, C3, and C4 respectively.

#### 3.3.2. Enabling factors

The differences of economic situation, disease burden, health insurance, living conditions, degree of child support, family relationships, primary caregiver, informal support, and quality of community medical services between the four latent classes were statistically significant (*p* < 0.05), while the differences in the number of children, service attitude of community staff, infrastructure facilities, satisfaction with community resources and convenience of access to clinics were not statistically significant (*p* ≥ 0.05). *Post-hoc* comparisons revealed that C3 members had better economic status than those in the other three groups, while C2 members reported the lowest economic status, as previously suspected. C4 members scored significantly better than the other three classes on burden of illness, child support and quality of medical services. The family relationships score was highest for C1, followed by C4, C3 and C2. In terms of housing status, older people in C1, C2, and C4 were more likely to live with a spouse, whereas those in C3 were more likely to live alone or with their children. Moreover, C3 members were most likely to have a paid carer or part-time helper, while C1 were most likely to provide frequent informal support to their offspring or grandchildren. The full results are presented in Table 4.

**Table 4:** Enabling factors of latent classes (*N* = 382).

Variables	C1^a^ (*n* = 115)	C2^b^ (*n* = 88)	C3^c^ (*n* = 100)	C4^d^ (*n* = 79)	*x*^2^/F	Scheffe *post-hoc*
Economic situation	3.50 ± 0.862	3.18 ± 0.924	3.60 ± 0.953	3.32 ± 0.825	4.344^**^	c > a > d, c > a > b
Disease burden	3.68 ± 1.189	3.43 ± 1.220	3.21 ± 1.192	3.70 ± 1.113	3.702^*^	d > a > b, d > a > c
Health insurance					28.557^**^	–
Urban employees’ insurance	67 (58.3%)	56 (63.6%)	78 (78.0%)	49 (62.0%)		
Urban residents’ insurance	20 (17.4%)	16 (18.2%)	11 (11.0%)	16 (20.3%)		
New rural social endowment insurance	25 (21.7%)	11 (12.5%)	8 (8.0%)	9 (11.4%)		
Commercial medical insurance	3 (2.6%)	0 (0.0%)	0 (0.0%)	0 (0.0%)		
None	0 (0.0%)	5 (5.7%)	3 (3.0%)	5 (6.3%)		
Living conditions					34.771^**^	–
Living with children	23 (20.0%)	12 (13.6%)	29 (29.0%)	14 (17.7%)		
Living with spouse	30 (26.1%)	38 (43.2%)	25 (25.0%)	24 (30.4%)		
Living with children and spouse	29 (25.2%)	15 (17.0%)	6 (6.0%)	17 (21.5%)		
Living alone	26 (22.6%)	22 (25.0%)	36 (36.0%)	17 (21.5%)		
Other	7 (6.1%)	1 (1.1%)	4 (21.1%)	7 (8.9%)		
Number of children					13.405	–
0	1 (0.9%)	2 (2.3%)	1 (1.0%)	1 (1.3%)		
1	30 (26.1%)	32 (36.4%)	23 (23.0%)	12 (15.2%)		
2	51 (44.3%)	29 (33.0%)	38 (38.0%)	36 (45.6%)		
≥3	33 (28.7%)	25 (28.4%)	38 (38.0%)	30 (38.0%)		
Degree of child support	4.22 ± 0.916	3.68 ± 1.225	4.10 ± 0.886	4.23 ± 0.878	6.111^***^	d > a > c, d > a > b
Family relationships	4.37 ± 0.705	3.80 ± 1.126	4.04 ± 0.803	4.22 ± 0.827	7.777^***^	a > d > c, a > d > b
Primary caregiver					43.058^***^	–
Self	77 (67.0%)	58 (65.9%)	43 (43.0%)	54 (68.4%)		
Spouse	12 (10.4%)	18 (20.5%)	13 (13.0%)	6 (7.6%)		
Children	12 (10.4%)	3 (3.4%)	13 (13.0%)	8 (10.1%)		
Other relatives or friends	0 (0.0%)	3 (3.4%)	1 (1.0%)	0 (0.0%)		
Nanny or hourly worker	14 (12.2%)	6 (6.8%)	30 (30.0%)	11 (13.9%)		
Informal support					26.917^***^	–
Never	26 (22.6%)	25 (28.4%)	46 (46.0%)	25 (31.6%)		
Occasional	30 (26.1%)	32 (36.4%)	32 (32.0%)	17 (21.5%)		
Often	59 (51.3%)	31 (35.2%)	22 (22.0%)	37 (46.8%)		
Service attitude of community staff	4.03 ± 0.898	3.75 ± 0.997	3.84 ± 0.735	3.99 ± 0.927	2.111	–
Quality of community medical services	3.29 ± 0.906	2.98 ± 0.907	3.15 ± 0.813	3.33 ± 0.839	2.915^*^	d > a > c, d > a > b
Infrastructural facilities	3.51 ± 1.038	3.30 ± 0.973	3.38 ± 0.838	3.58 ± 0.871	1.664	–
Satisfaction with resources	3.42 ± 1.068	3.31 ± 0.939	3.36 ± 0.823	3.52 ± 0.904	0.775	–
Convenience of access to clinics	3.75 ± 1.227	3.55 ± 1.286	3.65 ± 0.999	4.00 ± 1.074	2.356	–

*, **, and *** denote significance levels of 5, 1, and 0.1%, respectively. C1, high health and social interaction demand; C2, high comprehensive demand; C3, high care service demand; C4, high social participation and low care demand. ^a–d^denote C1, C2, C3, and C4 respectively.

#### 3.3.3. Need and perception of aging factors

There were statistically significant differences in the self-assessed health status, self-assessed mental status, number of chronic illnesses, satisfaction with the state of pension, degree of impaired capacity and acceptability of community social care rather than family care among the four different latent classes (*p* < 0.05). *Post-hoc* comparisons showed that C1 members had the highest self-rated mental health and capability, while C4 members had the highest scores for self-rated health and satisfaction with state of pension among the four classes. Regarding chronic diseases, the proportion of older people with one chronic disease was greater in C1, while the proportions with three and four or more chronic diseases were greatest in C3. For perception of aging, C3 members were most likely to accept community social care, whereas C4 members were least likely. Table 5 presents the full results.

**Table 5:** Need and perception of aging factors of latent classes (*N* = 382).

	C1^a^ (*n* = 115)	C2^b^ (*n* = 88)	C3^c^ (*n* = 100)	C4^d^ (*n* = 79)	*x*^2^/F	Scheffe *post-hoc*
Self-assessed health status	3.35 ± 1.009	3.07 ± 1.081	2.93 ± 0.987	3.62 ± 0.881	8.382^***^	d > a > b, d > a > c
Self-assessed mental status	4.03 ± 0.794	3.61 ± 1.108	3.63 ± 0.917	4.01 ± 0.824	6.230^***^	a > d > c, a > d > b
Number of chronic diseases					37.336^***^	–
0	16 (13.9%)	16 (18.2%)	4 (4.0%)	18 (22.8%)		
1	40 (34.8%)	26 (29.5%)	22 (22.0%)	30 (38.0%)		
2	27 (23.5%)	20 (22.7%)	23 (23.0%)	17 (21.5%)		
3	17 (14.8%)	15 (17.0%)	21 (21.0%)	8 (10.1%)		
≥4	15 (13.0%)	11 (12.5%)	30 (30.0%)	6 (1.6%)		
Satisfaction with state of pension	3.90 ± 0.688	3.47 ± 1.039	3.66 ± 0.855	3.99 ± 0.742	7.220^***^	d > a > c, d > a > b
Degree of impaired capacity	3.49 ± 0.640	3.38 ± 0.807	2.85 ± 0.716	3.48 ± 0.638	18.535^***^	a > d > b, a > d > c
Acceptability of community social care (rather than family care)	3.63 ± 1.072	3.75 ± 1.157	3.94 ± 0.930	3.42 ± 1.183	3.672^*^	c > b > a, c > a > d

*, **, and *** denote significance levels of 5, 1, and 0.1%, respectively. C1, high health and social interaction demand; C2, high comprehensive demand; C3, high care service demand; C4, high social participation and low care demand. ^a–d^denote C1, C2, C3, and C4 respectively.

### 3.4. Multinomial logistic regression analyses

An unordered multinomial, unconditional logistic regression analysis was conducted using the classification results of latent classes as the dependent variables and the variables with significant differences in the chi-square and one-way analysis of variance as the independent variables (*n* = 22), with C4 (high social participation and low care demand) as the reference class. The results showed that 12 independent variables had significant differences between the four latent classes (*p* < 0.05): knowledge of integrated care, willingness to pay for integrated care, economic situation, disease burden, quality of community medical services, self-assessed health status, gender, health insurance, living conditions, primary caregiver, informal support, and degree of impaired capacity. Table 6 presents the full results.

**Table 6:** Multinomial logistic regression analysis (*N* = 382).

Indicators	Variables	C1/C4		C2/C4		C3/C4	
		Exp(B)	SE	Exp(B)	SE	Exp(B)	SE
Predisposing factors	Male (female)	0.311^**^	0.406	0.335^*^	0.449	0.529	0.456
	Knowledge of integrated care	1.192	0.162	1.251	0.186	1.500^*^	0.186
	Willingness to pay for integrated care	1.163	0.217	2.049^**^	0.260	1.335	0.267
Enabling factors	Economic situation	1.330	0.257	0.756	0.294	2.058^*^	0.292
	Disease burden	1.238	0.201	1.220^*^	0.231	0.710	0.238
	**Health insurance (none)**						
	Urban employees’ insurance	2.215^***^	0.628	7.162^*^	1.009	4.266	1.090
	Urban residents’ insurance	3.013^***^	0.627	6.587	1.066	4.720	1.168
	New rural social endowment insurance	2.240	0.000	7.567	1.133	5.909	1.227
	Commercial medical insurance	1.408	2.494	2.078	0.000	8.957	0.000
	**Living conditions (other)**						
	Living with children	4.177	0.945	20.363^*^	1.534	4.545^***^	1.047
	Living with spouse	4.804	1.085	14.669	1.616	9.662^*^	1.140
	Living with children and spouse	5.687	1.098	7.571	1.658	10.644	1.230
	Living alone	5.388	0.836	5.990^**^	1.432	14.955^**^	0.923
	**Primary caregiver (nanny or hourly worker)**						
	Self	1.413	0.663	1.583	0.818	0.313	0.669
	Spouse	3.356	0.951	12.142^*^	1.054	1.298	0.972
	Children	1.122	0.850	0.530	1.127	0.316	0.880
	Other relatives or friends	11.730	0.000	2.548	2.509	2.422	0.509
	**Informal support (often)**						
	Never	0.487	0.501	0.591	0.565	2.731^*^	0.552
	Occasional	0.686	0.460	1.146	0.523	1.886^*^	0.524
	Quality of community medical services	0.890	0.221	0.547^*^	0.253	0.601^*^	0.257
Need factors	Self-assessed health status	0.505^**^	0.263	0.376^**^	0.301	0.756	0.281
	**Degree of capacity impairment (unimpaired)**						
	Heavily impaired	0.728	1.569	3.760	1.362	8.699	1.350
	Moderately impaired	0.810	0.943	0.462	1.131	16.829^**^	0.995
	Slightly impaired	0.590	0.496	0.714	0.567	5.307^*^	0.583

C1, high health and social interaction demand; C2, high comprehensive demand; C3, high care service demand; C4, high social participation and low care demand. C4 is the reference class. Reference variables are in brackets. *, ** and *** denote significance levels of 5, 1, and 0.1%, respectively.

In the C1/C4 model, men were 0.311 times more likely than women to be in C1. Older people who had basic medical insurance for urban workers or residents were more likely to be in C1 than C4. For every point increase in self-rated health status, older people become 0.505 times more likely to be in C4, which means that those with better self-rated health were less likely to be in C1.

In the C2/C4 model, men were more likely than women to be in C2. For every unit increase in willingness to pay for integrated services, the probability of being in C2 increased by a factor of 2.049. Moreover, the probability of being in C2 increased 1.220 times for every point increase in the burden of disease. Older people with urban employee health insurance were more likely to be in C2 than those without any health security. Older people whose primary caregiver was their spouse were 12.142 times more likely to be in C2 than those whose caregiver was a paid carer or hourly worker. Conversely, those who perceived that community medical services were high quality were less likely to be in C2, and older people with better self-rated health status were more likely to be in C4.

In the C3/C4 model, the probability of older people being in C3 increased as knowledge of integrated services rose. Older people who rated themselves as better off were also more likely to be in C3. Compared to those with other living situations, older people living with children, with their spouses, and living alone were, respectively, 4.545, 9.662, and 14.955 times more likely to be in C3. For every unit increase in the perceived quality of community medical services, an individual was 0.601 times more likely to be in C3 (i.e., higher perceived quality raised the likelihood of being in C4). Finally, older people with moderate or mild impairment (objectively assessed) were, respectively, 16.829 and 5.307 times more likely to be in C3, compared to unimpaired older people.

## 4. Discussion

Based on a sample of 382 older people in Changsha, Hunan Province, this study identified four latent classes of older people’s demand for home-and community-based integrated care and the predictors of those four latent classes Our findings deepen understanding of heterogeneous and individualized demand from older people and provide important evidence for designing new care sub-models and developing integrated care in China.

Through analyzing the diverse social and health problems experienced by older people, this study found that survey participants could be divided into four classes: high health and social interaction demand (C1), high comprehensive demand (C2), high care service demand (C3), and high social participation and low care demand (C4). In terms of similarities, C1 and C4 had similarly high social participation demand, while C2 and C3 both had higher demand for medical–nursing services. In terms of differences, C1 had the highest scores for social participation services and health education services. C2 had higher scores for all seven dimensions than other classes, and the highest scores of all four classes for psycho-spiritual support services and social aid services. C3 had the highest scores for basic life services and medical–nursing services, and the lowest score for social participation services. C4 was mainly characterized by high demand for social participation services and relatively low demand for the other six services, indicating that C4 members primarily required more social interaction and self-fulfillment opportunities.

Interestingly, there are three main similarities between this study’s findings and the results of Wang’s (19) LCA of the needs of older people using data from the 2014 Chinese Longitudinal Aging Social Survey. First, both studies identified four latent classes of older adults. Second, two of the four classes in both studies had higher need of medical care services. Wang’s equivalents for this study’s high care service demand (C3) and high comprehensive demand (C2) were described as “high health care demand” and “comprehensive high demand.” Third, Wang’s “healthy multi-demand” type had a high level of loneliness and the “low demand” type had low scores for all but one service (living-care), both of which are similar to the demand for more social participation only in this study’s C4. Although Wang’s study did not specifically focus on home-and community-based integrated care, but rather analyzed simpler dimensions and indicators compared to our study, she did cover the four major needs: living care, economic security, spiritual comfort and health care, which are similar to this study’s indicators. The similar findings of the two studies suggest that the current study’s insights are generalizable beyond Changsha and Hunan, as Wang’s study sample comprised 6,364 older people (aged 60 and over) from across China.

According to these four classes, different sub-models of home-and community-based integrated care can be explored for older people, while older people can change their choice of sub-model as their needs evolve. Firstly, according to the research, University of the Third Age (U3A) activities have a positive impact for older adults on health and quality of life (46), and older adults can self-manage as they live with their condition on a daily basis and develop strategies to look after themselves (47). To meet the high demand for social participation services, health education services and rehabilitation services for C1 members, a sub-model combining U3A with health-related activity (such as self-management, exercising, maintaining a healthy diet and so on) (48) could be tested. The former could meet the needs for health knowledge and cultural recreation, while the latter could meet older people’s physical and mental health needs.

Secondly, C2 members may benefit from a sub-model of “1+3,” with “1” denoting a family doctor and “3” the combination of a home-and community-based care service center, a primary community hospital, and a social work organization. This sub-model would allow older people who are able to move independently to seek help from community service centers. It would also meet the multiple needs of older people with multiple chronic illnesses and limited mobility through home health services provided by family doctors and social aid services led by the government in collaboration with social work organizations.

Thirdly, our study found that C3 had the highest proportion of people with multiple chronic diseases. Studies have shown that having chronic disease is related to demand for health services and that the number of chronic diseases influences the level of demand (49). To meet the high demands of C3 members, a suitable sub-model of “primary community hospitals contracted with high-level general hospitals + integrated care clinical pharmacist (ICP)” could be implemented. Both primary community hospitals and high-level general hospitals need to support and cooperate with the home-and community-based care service center by providing services such as voluntary medical consultations, home care and regular medical check-ups, thus ensuring an effective interface between health and nursing. High-level general hospitals could accept older patients from the community through the “two-way referral” in a timely manner to avoid delaying treatment and problems such as lack of capacity and poor quality of care in community health services (50). In addition, ICP is an innovative role, involving work in integrated teams, and receiving referrals from community nurses, general practitioners, geriatricians and other doctors, supporting frail older people in the community who need help in getting the best out of taking their medication (51).

For C4, two alternative sub-models of care may be suitable. The combination of U3A with HRA, as recommended for C1, may also be suitable for C4 members. However, the primary need in C4 is for social participation. Members are in good health, have low demand for nursing, and are suitable for the traditional family support model. On these bases, a sub-model combining education and entertainment with mutual help could be more appropriate. This would help older people find suitable cultural and recreational activities in the community, while the organization of more volunteer services (such as taking care of other seriously ill older people in the community, mediating disputes, donating money or goods to people in poverty, participating in health promotion and education activities in the community) would provide more opportunities to interact with others and to find personal value.

As older people exist in multiple social, family and personal environments, their demands for health and social care services are inevitably influenced by a combination of factors (29). The one-way ANOVA and multinomial regression analyses based on the extended ABMHSU in this study revealed a great degree of variation in the influencing factors across the latent classes. The findings indicate that older adults from various groups consider four key factors—predisposition, enabling, need, and perception of aging—when deciding whether to use integrated care services in their homes and communities. These dimensions can inform policy and health service research, helping to improve use behavior and revamp service delivery systems. Although the perception of aging factor did not meet the criteria for significance in the multinomial regression analysis, it is worth noting that participants in C3 were more open to receiving integrated care services beyond the traditional family caregiving model compared to those in the other three groups, especially C4.

Among the predisposing factors, men were more likely to be in C4 (greater social participation needs). This may be explained by women taking on greater responsibility for intergenerational support and household activities, even in retirement (52). Another plausible explanation is that men are relatively advantaged in terms of literacy, social participation, and the distribution and appropriation of social resources (53). However, some studies using Andersen’s model have shown that gender does not significantly influence older people’s choice of home-and community-based medical care and aging services (30). The willingness of older people to pay for integrated care is directly proportional to their probability of being in C2. That is, older people’s willingness to pay affects their comprehensive demand for home-and community-based medical care (as all seven dimensions of services are in demand by members in C2). The pilot Qualitative Study in China on Community-Based Adult Services (CBAS) integrated care also found that older people who could benefit did not use these services because of limitations of “economic capability” and “willingness to pay” (9). Therefore, to ensure that home-and community-based integrated care is used effectively and truly meet the needs of older people, solutions and pathways for reimbursement or reduction of costs should be devised.

As knowledge of integrated services increases, the probability of older people being in C3 increases. The economic status of people in C3 was better than the other three groups, they were more likely to live alone or with their children (but not very close to them), and they had a higher probability of having chronic and multiple chronic illnesses. More importantly, they were more open-minded and receptive to community social care than the other three groups of older people. So, as they become more aware of the benefits of integrated care, their needs increase accordingly, and it can be argued that knowledge makes the underlying need for care more explicit. Consistent with previous studies, the more older people know about integrated care, the higher their demand for basic living services and medical care (18). The insufficient understanding of integrated care among older people can easily lead to a decrease in their participation, resulting in the phenomenon of care resource idleness or waste in communities with relatively well-equipped care services and facilities. Some older people may not even be aware of the care services available on their doorstep, and if their families are unable to meet their care needs, they may be forced to leave their familiar social circle and live in nursing homes (9). It is also necessary to vigorously promote and publicize information about integrated care, to increase older people’s awareness of, and engagement with, these services, while avoiding any unnecessary use of community resources.

Among the enabling factors, and compared to C4, older people who had urban employees’ insurance or urban residents’ insurance were more likely to be in C1, while those who had a higher disease burden or lived alone were more likely to be in C2. Considering the welfare and security of older people and the serious disease burden they may experience when suffering from multiple chronic diseases, it is necessary to continue to promote the piloting of long-term care insurance (LTCI) in more cities in China. This could alleviate financial pressures through subsidies for care costs and free care services (19). The perceived quality of community medical services is inversely related to the probability of older people being in C2 and C3. Previous studies also found that many older people complained that their communities did not provide adequate and satisfactory care, and even felt they could not trust the existing community infrastructure and level of medical care staff ‘s expertise (9). This calls for a focus on the high quality and availability of health services in the community, especially for older people with multiple chronic illnesses and high care needs, such as those in C2 or C3, who have difficulty meeting some of their urgent care needs when the quality of medical services in the community are low.

Among the need factors, older people with higher self-rated health were less likely to be in C1 or C2 and more likely to be in C4, while those with slightly or moderately impaired abilities were more likely to be in C3 than C4. Overall, those in C4 had better health and could take care of themselves and their spouses. Their greatest need was for more social interaction and cultural and recreational services in the community.

It is important to recognize that there is no internationally understood and generally accepted English translation of *Yiyang Jiehe*, which leads to misunderstandings and to difficulties in generating academic consensus in the international community about research on this topic in China. Another key consideration is that the overall path of China’s integrated care development seems to be bottom-up—the series of state publications on *Yiyang Jiehe* are indicative of piecemeal progress, with no standardized, detailed and complete policy documents on home-and community-based integrated care (28, 54). Different models of this type of care are being piloted in different provinces and municipalities (19), and a complete system of *Yiyang Jiehe* in the home and community has yet to be formed. To protect the legal rights and health equity of older adults, and to clarify the obligations and responsibilities between community service providers and older people, China must legislate on standards for home-and community-based integrated care services, drawing on the program-based experience of Western countries. For example, the Community-Based Adult Services program introduced by the California Department of Aging in 2012 established clear laws and regulations, regulates the content and standards of services, is linked to relevant health insurance programs (e.g., Medicare) and the Program of All-Inclusive Care for the Elderly (PACE), and provides a dedicated website for citizens to learn about and access details of the service (55).

As the highest proportion of surveyed older people was in C1 (115, 30%), characterized by high demand for social interaction and medical care, it is necessary to incorporate the “active aging” concept (56) into China’s home-and community-based integrated care services. Promoting this concept in China will not only be conducive to guiding society, communities and older people to view aging as an active, positive and dynamic process, it will encourage them to participate in the shared governance of community aged services, and enhance older people’s confidence in embracing society and realizing their values in life.

To the best of the authors’ knowledge, this study is among the first to analyze latent classes of demand for home-and community-based integrated care and its predictors in China. However, several limitations must be considered in interpreting the findings. First, this paper focuses only on Changsha City, Hunan, and the relatively limited sample size may affect the generalizability of its results. Second, the target survey population was limited to older people, so the study lacks insights from their caregivers and family members and from the staff of community care service centers. Future studies should update and refine the tools and methods for assessing integrated care demands, conduct longitudinal analysis, and include more provinces and cities in China to build on our findings. The perspectives of caregivers are also important, as some older people with dementia or disability may not be able to express their demands independently and comprehensively, whereas their caregivers can reflect their needs to some extent (9).

## 5. Conclusion

In summary, this study constructed a demand assessment tool for home-and community-based integrated care for older adults through a rigorous research process based on an extended ABMHSU. It explored different latent classes of demand for home-and community-based integrated care in Chinese older adults and the factors that influence these different classes. Four innovative types of home-and community-based integrated care services were also proposed based on different classes, which help to increase the level of demand for integrated health care services and improve the overall level of protection of the community. The findings suggest that home-and community-based integrated care involves multiple systems, various stakeholders, and collaboration among different units and departments. Besides developing service platforms, differentiated integrated care services must be provided to meet the heterogeneous needs of older people. Only then can we achieve a positive and deep development of Y*iyang Jiehe* in China. Finally, the findings from this study may be useful for southeast Asian countries or other developing countries, where there may be some commonalities in the needs of older people. However, as models of care for older people vary from country to country, the choice of sub-model of care for local older people will need to be determined on a case-by-case basis.

## Data availability statement

The original contributions presented in the study are included in the article/Supplementary material, further inquiries can be directed to the corresponding author.

## Ethics statement

The study was approved by the Clinical Medical Ethics Committee of Xiangya Hospital, Central South University (No. 202011184). Written informed consent was obtained from participants in accord with the national legislation and institutional requirements.

## Author contributions

ZW and ZL conceived and designed the study. ZW collected data, processed statistics, analyzed results, and wrote and revised the article. ZL was responsible for proofreading the article and for funding acquisition. All authors contributed to the article and approved the submitted version.

## Funding

This research was funded by the Project of National Natural Science Foundation of China (71603289); Project of Hunan Provincial Natural Science Foundation (2022JJ30055); Project of Hunan Provincial Social Science Achievements Review Committee (XSP22ZDI008); Project of Changsha Science and Technology Plan (kh2302039).

## Conflict of interest

The authors declare that the research was conducted in the absence of any commercial or financial relationships that could be construed as a potential conflict of interest.

## Publisher’s note

All claims expressed in this article are solely those of the authors and do not necessarily represent those of their affiliated organizations, or those of the publisher, the editors and the reviewers. Any product that may be evaluated in this article, or claim that may be made by its manufacturer, is not guaranteed or endorsed by the publisher.
